# Interactions with the MC4R rs17782313 variant, mental stress and energy intake and the risk of obesity in Genome Epidemiology Study

**DOI:** 10.1186/s12986-016-0096-8

**Published:** 2016-05-21

**Authors:** Sunmin Park, James W. Daily, Xin Zhang, Hyun Seok Jin, Hye Ja Lee, Yong Hyun Lee

**Affiliations:** Department of Food and Nutrition, Obesity/Diabetes Research Center, Hoseo University, 165 Sechul-Ri, BaeBang-Yup, Asan-Si, Chung Nam-Do 336-795 South Korea; Department of R&D, Daily Manufacturing Inc., Rockwell, NC USA; Department of Biomedical Science, Hoseo University, Asan, South Korea; Center for Biomedical Science, Korea National Institute of Health, Cheongju, South Korea; Department of Nanobiomechatronics, Hoseo University, Asan, South Korea

**Keywords:** Melanocortin-4 receptor, Genotype, Korean Genome Epidemiology Study, Food preference, Dietary intake

## Abstract

**Background:**

The melanocortin-4 receptor (MC4R) regulates metabolism by modulating eating behavior and MC4R variants (rs17782313 and rs571312) are associated with obesity in Asians and Caucasians. However, the impact of their interactions with nutritional and lifestyle factors on obesity are poorly described. Therefore, we investigated the interaction of MC4R variants and dietary patterns on the risk of obesity in Korean middle-aged adults.

**Methods:**

Data collected included, genetic variations, anthropometric and biochemical measurements, dietary and lifestyle habits, and food intake. Data were obtained from the 8830 adults aged 40–69 years in the Ansung and Ansan cohort of the Korean Genome Epidemiology Study.

**Results:**

The MC4R rs18882313 minor allele had a higher frequency in the obese group (*P* < 0.01). MC4R genotypes were not associated with differences in daily energy and macronutrient intakes. However, the intakes of processed foods and fat (as percentages of energy) were significantly higher and intake of fruits were significantly lower in subjects with MC4R minor alleles (*P* < 0.05). Interestingly, there was a positive interaction between MC4R variants and mental stress levels that were associated with the risk of obesity after adjusting for age, gender, residence area, daily energy intake, smoking status and physical activity (interaction *P* = 0.0384). Only in subjects with high stress were MC4R minor alleles associated with higher BMIs after adjusting for confounders. The association was present without modulating energy and nutrient intake. In the group with energy intakes higher than estimated energy requirement (EER), subjects with MC4R minor alleles had higher BMIs than those with the major alleles (*P* < 0.001).

**Conclusions:**

The interactions of mental stress and energy intakes with the MC4R minor allele genotype might be associated with increased risk of obesity in Korean adults. This research might identify subjects with a specific MC4R minor alleles as a human subset of people with a low metabolic tolerance for excessive energy intake, especially when under stress.

## Background

Obesity is a significant health challenge worldwide and has negative impacts on health, from reducing life expectancy to increasing the risks of several diseases. It is believed that the main reasons for the increase in obesity in the last 40 years are changes in lifestyle and food supply, behavioral factors such as physical inactivity, and unhealthy diets including more processed foods. However, obesity is also caused by interactions among genetic variants, and is highly heritable [1].

Genome-wide association studies (GWAS) are powerful tools for discovering genetic variants associated with obesity and other diseases [2, 3]. The first gene discovered to have variants associated with obesity was fat mass and obesity-associated protein (FTO) [4]. Other genes associated with obesity have since been reported, including the melanocortin-4 receptor (MC4R) [5], adiponectin, C1Q and collagen domain containing [6], brain-derived neurotrophic factor [7], leptin [8], peroxisome proliferator-activated receptor gamma-2 [9] and SH2B1 genes [10]. Among these genes, some single-nucleotide polymorphisms (SNPs) near MC4R (rs17782313, rs571312, rs17700144, and rs2331841) are strongly associated with obesity in adults, adolescents, and children, indicating that subjects with minor alleles of these SNPs typically exhibit higher BMIs than those with the major allele [3, 11–13]. An association between MC4R rs17782313 and obesity has been reported in a European population [11, 14]. There was also a strong association between MC4R rs17782313 polymorphisms in subjects either heterozygous or homozygous for the C allele (CT or CC as opposed to TT) and higher body mass index (BMI) in Tatar women and Chinese people [14, 15]. In Korea, the MC4R variant rs17782313 was associated with BMI in a replication gene association study and a GWAS [16, 17] and BMI increased with C allele (minor allele) of MC4R rs17782313 by 0.22 kg/m^2^ BMI [17]. Subjects with the minor allele of MC4R rs17782313 exhibited a positive association with BMI and it tended to be related to a positive energy balance with possible impacts on dietary intake [18].

MC4R is expressed in regions of the central nervous system, including the hypothalamus, cerebral cortex, brain stem, and spinal cord [18]. MC4R is a component of the leptin system, which regulates energy intake with neuropeptide effectors such as pro-opiomelanocortin (POMC), α–melanocyte-stimulating hormone (α-MSH), and agouti-related peptide (AGRP) [19]. When the body is in a negative energy state, the decrease in leptin levels leads to lower POMC expression, which reduces α-MSH levels, simultaneously stimulating the expression of AGRP in the orexigenic neurons of the arcuate nucleus, which is an antagonist at the MC4R [19, 20]. The decrease in α-MSH and increase in AGRP, and subsequent sustained repression of MC4R, result in increased food intake, which may cause obesity [18, 19].

MC4R polymorphisms may be associated with lifestyle, food intake, dietary habits, and specific nutrient preference [18, 21, 22]; however, this is still controversial. A study in Europeans indicated that MC4R rs17782313 is associated with higher BMI and overeating behaviors [18, 21]. In Iranian adults, the MC4R rs17782313 variant is related to high energy intake and low intakes of carbohydrates and protein [22]. However, Hassellbalch et al. [23] has reported that MC4R genotypes do not influence dietary intake. In addition, accumulating evidence suggests a functional interaction between MC4R and stress response [24]. Acute emotional stress activates POMC and its derivative α-MSH, which then increases the level of MC4R. MC4R activates the hypothalamic-pituitary-adrenal (HPA) axis and adrenocorticotropic hormone (ACTH), which increase the production and release of cortisol in response to stress [25, 26]. Mental stress also activates the HPA axis: it immediately induces a corticotropin-releasing-hormone-mediated suppression of food intake and chronically elevated glucocorticoids result in chronically stimulated eating behavior and weight gain [27]. Mental stress is known to increase the appetite for highly palatable and high-energy foods [28, 29]. Stress-induced activation of the HPA axis is attenuated in a rat model of MC4R loss-of-function [26]. MC4R facilitates an increase in anxiety-like and depression-like behaviors pursuant to chronic stress [30]. Thus, the MC4R polymorphism and stress may interactively change eating behavior leading to overweight and obesity. However, no study has been conducted to determine the interaction of MC4R polymorphisms and mental stress and nutrient intake.

Because MC4R is involved in eating behavior and stress and MC4R variants are associated with obesity, MC4R variants may modulate energy balance via gene-nutrient interactions. We hypothesized that MC4R variants affect body weight by modulating eating behavior and stress responses. To investigate this, we determined the interaction between the MC4R variant rs17782313 and both nutrient intake and mental stress in the development of overweight and obesity among 8842 Korea adults over 40 years of age from the Korean Genome Epidemiology Study (KoGES) study.

## Methods

### Subjects

The data collected in 2001 from subjects of the Ansung and Ansan cohort of the KoGES were used in this study [16]. Briefly, the participants were recruited from two community-based epidemiological cohorts: the rural community of Ansung city and the urban community of Ansan city. A total of 8842 subjects (4183 men and 4659 women; age, 40–69 years) participated. This study was approved by the institutional review board of the Korean National Institute of Health for the KoGES and Hoseo University. Written informed consent was obtained from all of the subjects.

### Basic characteristics and biochemical measurements

All of the participants had resided within the survey area for at least 6 months, and were mentally and physically healthy. Information on age, education, income, smoking history and alcohol consumption, and overall activity were collected during a health interview.

Parameters measured were height, weight, BMI (weight [kg]/square of height [m^2^]), waist and hip circumference, waist-to-hip ratio, and blood pressure. Obesity was defined as BMI ≥ 25. Percent body fat (%BF) was measured by tetrapolar bioelectrical impedance analysis (Inbody 3.0, Biospace, Seoul, Korea). Previously validated, empirically derived formulas were used for bioelectrical impedance analysis of fat and lean tissue [31]. Education level was categorized into three groups: less than high school, high school, and college or more. Household income (USD/month) was divided into four groups: very low (<1000), low (1000–2000), intermediate (2000–4000), and high (>4000). Smoking status was divided into three categories: current smoker, past smoker, and never smoker. Alcohol consumption was assessed by questioning the participants about their drinking behavior during the month prior to the interview. Alcohol consumption status was divided into four groups according to average daily consumption (g/week): nondrinker, moderate drinker (1–25), and heavy drinker (>25). Total activity was categorized into “little”, “moderate” and “heavy” physical activity. Moderate activity was considered as the participation in moderate exercise (slow swimming, doubles tennis, volleyball, or occupational or recreational activity involving the carrying of light objects) for ≥ 30 min at a time three times per week, or as participation in vigorous exercise (running, climbing, fast cycling, fast swimming, football, basketball, rope jumping, squash, singles tennis, or occupational or recreational activity involving the carrying of heavy objects) for ≥ 20 min at a time once per week. Subjects were considered as having little activity if they had less than moderate activity whereas those with more than moderate activity were categorized into the heavy activity group. Mental stress was evaluated by asking subjects to 10 questions concerning their state of agitation and anxiety in the workplace and family situations in their daily life. The questions are as following: 1) Have a stomachache with anxiety and anger, 2) Be stressed in workplace and home, 3) Do eat, drink or smoke when being stressed, 4) Have headache, pain in neck and shoulders and insomnia, 5) Not relieved from stress by free time at night and weekends, 6) Not able to concentrate on routine life due to worrying about work, 7) Take a medicine to relive stress, 8) Hard to have enough time to relax, 9) Do not use free time enough to relieve stress and tension, and 10) Am pressed by deadlines at work. Each question about stress was answered with no (0), occasional (1) and frequent (2) stress. The severity of mental stress was calculated by the sum of all answers. Mental stress was categorized into three groups such as mild stress (<2), moderate stress (2–5) and severe stress (>6).

### Genotyping and quality control

The genotype data were graciously provided by the Center for Genome Science, Korea National Institute of Health. The detailed genotyping and quality-control processes were previously described [16]. Briefly, most DNA samples were isolated from the peripheral blood of participants and genotyped using the Affymetrix Genome-Wide Human SNP array 5.0 (Affymetrix, Santa Clara, CA). The accuracy of the genotyping was examined using the Bayesian Robust Linear Modeling with Mahalanobis Distance genotyping algorithm [32]. Samples with low genotyping accuracies of < 98 %, high missing genotype call rates (≥4 %), high heterozygosity (>30 %), or gender biases were excluded.

### Assessment of foods and nutrient intake

The Korean dish-based semi-quantitative food frequency questionnaire (FFQ) was used to assess long-term food intake of the 8830 participants in the KARE studies. The validity and reproducibility of this FFQ were evaluated by previous studies in Korean population [33, 34]. This FFQ demonstrated moderate correspondence with the four 3-day food records for 4 seasons as tailing to 12-day food records in previous studies. Adjusted correlation coefficients between FFQ and 12-day food records ranged between 0.23 and 0.64 and the validation and reproducibility of this FFQ were acceptable [33]. This questionnaire requested information regarding the participant’s average consumption of food items during last l year. The FFQ included 103 food items and the intake of food frequencies was divided into nine categories: never or seldom, once a month, two to three times a month, one to two times a week, three to four times a week, five to six times a week, once a day, twice a day, and three times or more every day. The amount of food intake at once was checked as “more”, “equal”, or “less” on the basis of the portion size. FFQ data were converted into food intake per day in each food category by multiplying the number of times each food was consumed by the amount of food intake. The portion size was given by pictures of foods in each food category and participants selected the frequencies based on the defined portion size. The daily intake was computed based on the midpoint of the reported frequency category for each food item. For example, when one food item was checked as 2–4/week, and it was calculated to be 3/7 or 0.43 times/day.

Daily nutrient intake was calculated from semi-quantitative food frequency questionnaires. From the food intake, energy and nutrients such as protein, carbohydrates, fat, fiber, total vitamin A, vitamin C, Na, Ca, and K were calculated using the Can-Pro 2.0 nutrient intake assessment software developed by the Korean Nutrition Society. Daily estimated energy requirement (EER) and recommended nutrient intake were used from Korean dietary reference intake (KDRI) according to age and gender [35].

### Statistical analysis

Statistical analyses were performed using GPLINK version 2.0 (http://pngu.mgh.harvard.edu/~purcell/plink) and SAS (version 9.3; SAS Institute, Cary, NC, USA). The descriptive statistics of participants for categorical variables, such as gender and dietary habits, were obtained by determining frequency distributions. Frequency distributions by classification variables were analyzed using the Chi-squared test. The descriptive statistics of continuous variables are expressed as means with standard deviations (SDs). Multivariate adjustments for comparisons of continuous variables were carried out by generalized linear models. The results were adjusted for age, gender, and residence area. To examine the interaction between the MC4R rs17782313 variants and dietary patterns or lifestyles, separate multivariate regression models were used for including the corresponding main effects and interaction terms in addition to the potential confounders. Next, odds ratios (ORs) and 95 % confidence intervals (CI) for dietary habits and different food items were calculated using different genotypes of MC4R with controlling for covariates using multivariable logistic regression method. The confounders used for the analysis were age, gender, residence area, BMI, total energy intake, physical activity, and smoking status as indicated.

## Results

### Baseline characteristics of normal, overweight and obese subjects

The rates of overweight and obesity were higher as the subjects aged (*P* < 0.001). Women were more often obese than men (*P* < 0.01) and higher incomes were associated with overweight and obesity (*P* < 0.001; Table 1). Drinking was associated with higher rates of overweight and obesity but smoking was associated with lower rates of obesity (Table 1). As expected, physical activity was found to have a negative association with overweight and obesity (*P* < 0.001). However, mental stress was not independently associated with the rates of overweight and obesity. We observed statistical differences in genotype frequencies of MC4R polymorphism among the normal-weight, overweight and obesity groups (*p* < 0.01; Table 1). The frequencies of subjects with minor alleles were higher were in the ascending order of normal-weight, over-weight and obese groups.

**Table 1:** Baseline characteristics of subjects according to obesity

	Categories	Normal (BMI < 23)	Overweight (23 = <BMI < 25)	Obesity (BMI > =25)
Age	30–45	966 (41.0)	579 (24.6)	813 (34.5)
	45–65	2295 (43.0)	1124 (21.0)	1924 (36.0)
	65+	648 (56.8)	168 (14.7)	325 (18.5)^***^
Gender (male %)		1855 (44.4)	936 (20.1)	1669 (35.8)^**^
Income (USD/month)	<1000	1644 (53.8)	516 (16.9)	896 (29.3)
	1000 ~ 2000	1107 (24.8)	535 (21.2)	885 (35.0)
	2000 ~ 4000	899 (36.8)	614 (25.2)	928 (28.0)
	>4000	171 (26.0)	187 (28.4)	201 (45.7)^***^
Drinking (g/week)	No	2407 (44.9)	1095 (20.4)	1863 (34.7)
	Moderate (1 ~ 25 g)	917 (43.1)	488 (22.9)	725 (34.0)
	Heavy (>25 g)	446 (41.2)	238 (22.0)	398 (36.8)^*^
Smoking	Non	2216 (43.1)	1080 (21.0)	1847 (35.9)
	Past	523 (38.6)	307 (22.7)	524 (38.7)
	Current	1102 (49.5)	469 (21.1)	657 (29.5)^***^
Total activity	Little	1537 (37.7)	943 (23.1)	1597 (39.2)
	Moderate	831 (39.7)	496 (23.7)	767 (36.6)
	Heavy	1354 (58.0)	388 (16.6)	591 (25.3)^***^
Stress	A little	2652 (44.6)	1238 (20.8)	2058 (34.6)
	Moderate	1109 (43.3)	574 (22.4)	877 (34.3)
	Heavy	148 (44.3)	59 (17.7)	127 (38.0)
MC4R rs17782313	TT	2302 (59.0)	1389 (35.6)	214 (5.5)
	CT	1039 (55.6)	711 (38.0)	119 (6.4)
	CC	1692 (55.4)	1146 (37.5)	218 (7.1)^**^

N (row %)

^*^Significantly different in *χ*2 test at *P* < 0.05. ^**^ *P* < 0.01. ^***^ *P* < 0.001

### MC4R variants rs17782313 and rs571312 and BMI

**Fig. 1:**
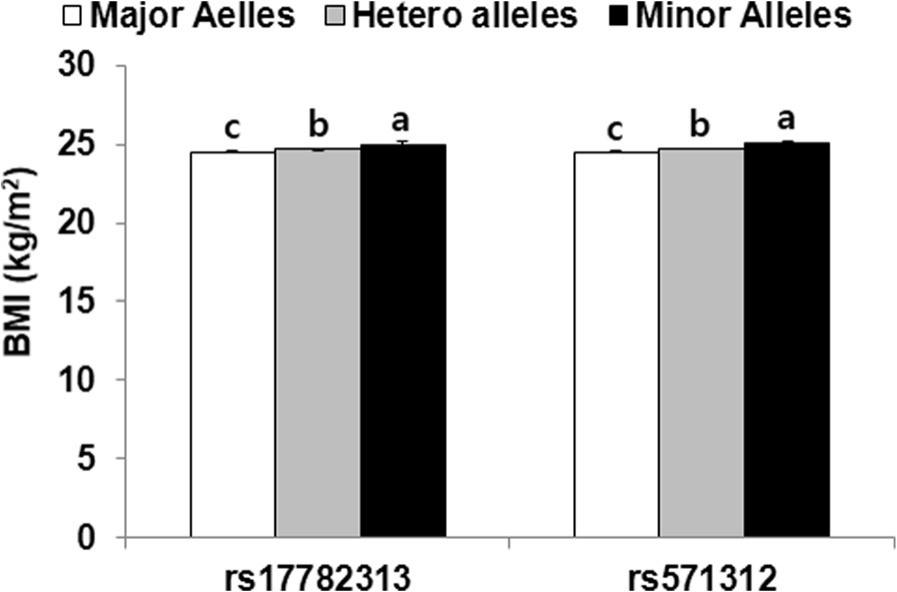
Body mass index (BMI) of melanocortin-4 receptor rs17782313 and rs571312 variants. Bars and error bars represented adjusted means and standard errors after adjusting for age, gender, residence area body mass index, daily energy intake, smoking status and physical activity. ^abc^ Different letters on the bars indicate significant differences at *P* < 0.05

The minor allele frequency (MAF) of MC4R rs17782313 was 25 %. The distribution of the MC4R rs17782313 genotypes was in Hardy-Weinberg equilibrium (*p* = 0.37). Both the minor alleles C of rs17782313 and A of rs571312 were significantly associated with BMI. BMI was significantly higher in the ascending order of major alleles, heterozygotes and minor alleles in both MC4R rs17782313 and MC4R rs571312 without (*p* = 0.0008) and with adjusting for confounders including age, gender, residence area, daily energy intake, total activity, and smoking status (*p* = 0.0012; Fig. 1).

### Nutrient intake according to MC4R genotypes

Daily intake of energy, carbohydrate, fat and protein was not significantly different among MC4R rs17782313 genotypes after adjusting for age, gender, residence area and BMI (Table 2). Additionally, the daily energy intake as a percentage of the estimated energy requirement (EER), and energy percentage of carbohydrate and protein did not show any significant differences among MC4R polymorphisms after adjusting for confounders (Table 2). However, energy percentage of fat was significantly different among the MC4R genotype groups after adjusting for confounders (*P* < 0.05; Table 2).

**Table 2:** Daily nutrient intake according to MC4R genotypes

	TT (*n* = 5033)	CT (*n* = 3246)	CC (*n* = 551)
Energy intake (kcal)	1915 ± 694^a^	1932 ± 733	1933 ± 839
CHO (g)	335 ± 114	339 ± 119	334 ± 135
Protein (g)	65.9 ± 31.1	66.4 ± 32.4	67.3 ± 33.5
Fat (g)	31.9 ± 21.2	32.0 ± 21.8	33.7 ± 25.1
Percentage of energy intake based on EER	91.6 ± 34.4	91.9 ± 32.5	92.8 ± 39.8
Percentage of CHO intake based on energy intake	70.8 ± 7.0	70.9 ± 6.8	70.1 ± 7.1
Percentage of protein intake based on energy intake	13.6 ± 2.4	13.6 ± 2.4	13.7 ± 2.4
Percentage of fat intake based on energy intake	14.4 ± 5.4*^,b^	14.3 ± 5.2*	14.9 ± 5.6*

EER, estimated energy requirement; CHO, carbohydrate

^a^Geometric means ± SD

^b^After adjusting for age, gender, residence area body mass index, daily energy intake, smoking status and physical activity

*Means with different letters indicated significant differences among the groups at *P* = 0.05

### Association between MC4R rs17782313 and food intake

We used the TT genotype as the reference to determine the adjusted OR for the relationship between MC4R rs17782313 and food intake after adjusting for confounders such as age, gender, residence area, daily energy intake, smoking status and physical activity (Table 3). MC4R rs17782313 C allele had a positive association with ramen and processed foods including canned tuna, fish cake, ham and cheese compared to the rs17782313 T allele after adjusting for confounders (OR = 1.021, 95 % CI = 1.004–1.038, *p* = 0.0173; OR = 1.010, 95 % CI = 1.001–1.019, *p* = 0.0254) (Table 3). Although the subjects with MC4R minor allele had higher intake of processed foods, they did not consume other high fat foods like bacon. MC4R minor alleles were negatively associated with the intake of fruits after adjusting for confounders (OR = 0.991, 95 % CI = 0.982–0.999, *p* = 0.0350) (Table 3).

**Table 3:** Adjusted odds ratios of food intake measured by food frequency questionnaires according to MC4R genotypes^a^

	TT (*n* = 5033)	CT (*n* = 3246)	CC (*n* = 551)
Rice	1^b^	1.001 (0.995–1.007)^c^	0.992 (0.979–1.004)
Cereals rice	1	0.998 (0.992–1.004)	0.993 (0.980–1.005)
Sweets^d^	1	0.999 (0.996–1.003)	1.002 (0.996–1.008)
Ramen	1	1.006 (0.974–1.040)	1.021 (1.004–1.038)*
Processed food^e^	1	1.006 (0.989–1.023)	1.010 (1.001–1.019)*
Beef	1	1.005 (0.984–1.027)	0.987 (0.984–1.027)
Pork	1	0.993 (0.975–1.010)	1.005 (0.971–1.040)
Bacon	1	0.991 (0.983–0.999)	1.004 (0.989–1.020)
Fish	1	1.012 (0.999–1.026)	1.014 (0.989–1.040)
Kimchi	1	0.998 (0.993–1.003)	0.999 (0.990–1.008)
Coffee	1	1.002 (0.999–1.004)	1.004 (0.998–1.009)
Tea	1	1.007 (0.997–1.017)	1.004 (0.985–1.024)
Milk and yogurt	1	1.001 (0.998–1.003)	1.004 (0.999–1.009)
Fruits	1	1.001 (0.997–1.005)	0.991 (0.982–0.999)*

^*^Significantly different from reference group at *P* = 0.05

^a^Adjusted by age, gender, residence area, body mass index, daily energy intake, smoking status and physical activity

^b^Reference group

^c^Odds ratio and 95 % confidence intervals

^d^Sweets included cake, candy, ice cream and chocolate

^e^Processed foods included canned tuna, fish cake, ham and cheese

### Interaction between MC4R rs17782313 and energy and fat intake to determine the risk of obesity

**Fig. 2:**
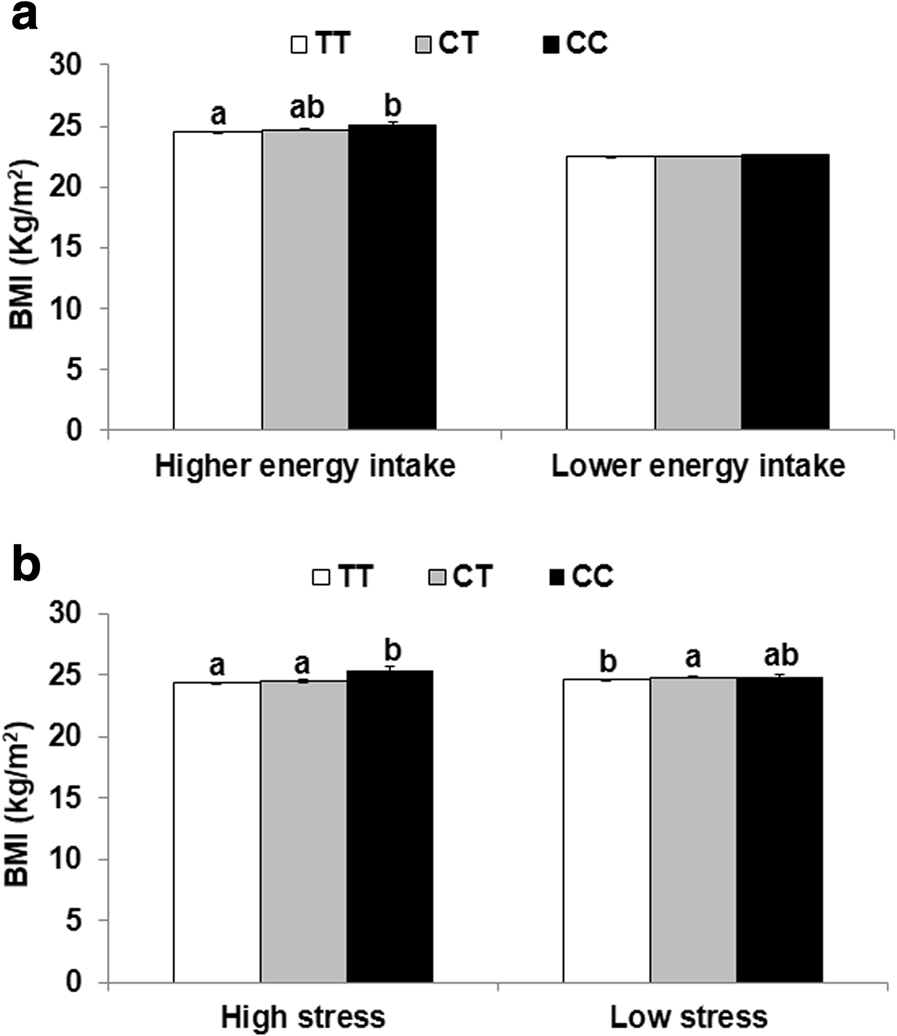
Body mass index (BMI) of MC4R rs17782313 genotypes according to energy intake and stress levels. A BMI according to energy intake. B BMI according to stress levels. Bars and error bars represented adjusted means and standard errors after adjusting for age, gender, residence area body mass index, daily energy intake, smoking status and physical activity. ^ab^ Different letters on the bars indicate significant differences at *P* < 0.05

There was relevant, but not quite significant (*P* = 0.594), interaction between energy intake and MC4R polymorphism in determining the risk of obesity after adjusting for confounders such as age, gender, area, total activity and smoking status (*P* = 0.0594; Table 4). According to these interactions, the association of MC4R genotypes and obesity was dependent on the energy intake. When energy intake was greater than EER, subjects with heterozygotes and minor had a higher risk of obesity (OR = 1.033, 95 % CI: 1.005–1.039 and OR = 1.053, 95 % CI: 1.019–1.087, respectively) (Table 4). In correspondence of association analysis, BMI was higher with ascending order of MC4R genotype TT, CT and CC in participants with energy intake higher than EER after adjusting for age, gender, area, total activity and smoking status (Fig. 2a). However, there was no association of MC4R polymorphisms with obesity as energy intake was lower than EER (Table 4) and BMI was not significantly different among the different groups of MC4R genotypes (Fig. 2a).

**Table 4:** Association between MC4R rs17782313 polymorphism and obesity according to energy and fat intake

		TT	CT	CC	Interaction^d^
Energy intake	Higher than EER	1^a^	1.022 (1.005–1.039)*^,b^	1.053 (1.019–1.087)**	0.0594
	Lower than EER	1	1.002 (0.945–1.062)	1.064 (0.961–1.177)	
Fat intake	Higher fat (≥14 %)	1	1.015 (0.989–1.042)^b^	1.052 (1.003–1.103)*	0.4531
	Lower fat (<14 %)	1	1.021 (0.996–1.047)	1.045 (0.994–1.099)	
Stress status	High stress	1	1.012 (0.983–1.042)^c^	1.112 (1.054–1.173)***	0.0359
	Low stress	1	1.027 (1.006–1.049)*	1.020 (0.978–1.062)	

^*^Significantly different from reference group at *P* = 0.05. ^**^ *P* = 0.01, ^***^ *P* = 0.001

^a^Reference group

^b^Odds ratio and 95 % confidence intervals after adjusted for age, gender, residence area, total activity and smoking status

^c^Odds ratio and 95 % confidence intervals after adjusted for age, gender, residence area, daily energy intake, total activity and smoking status

^d^ *P* value for the interaction term between MC4R polymorphism and energy intake, fat intake or stress status in the logistic regression model

We did not find a statistically significant interaction between fat intake and MC4R polymorphism in investigating the prevalence of obesity after adjusting for confounders such as age, gender, area, total activity and smoking status (*P* = 0.4531; Table 4). However, there was a significant association between obesity and fat intake and MC4R genotypes when fat intake was higher than 14 % of energy, which was the median of these cohorts. In the higher fat intake group, subjects with minor alleles had a higher risk of obesity (OR = 1.052, 95 % CI: 1.003–1.103; Table 2) (Table 4). Subjects with lower fat intake did not show any association with MC4R genotypes and the risk of obesity. The intake of carbohydrates and proteins did not exhibit any significant association with MC4R genotypes and the risk of obesity (data not shown).

### Interaction between MC4R rs17782313 and mental stress is associated with the risk of obesity

There was a significant and relevant interaction between mental stress levels and MC4R polymorphism when determining the risk of obesity after adjusting for confounders such as age, gender, area, daily energy intake, total activity and smoking status (*P* = 0.0359; Table 4). In subjects with high stress levels, those with minor alleles had a higher risk of obesity (OR = 1.112, 95 % CI: 1.054–1.173; *P* < 0.001) (Table 4). However, in subjects with low stress, heterozygotes for the minor allele were also found to have a higher risk of obesity (OR = 1.027; 95 % CI: 1.006–1.049; *P* = 0.0105) (Table 4). In correspondence of association analysis, BMI was higher with ascending order of MC4R genotype TT, CT and CC in participants with high stress levels after adjusting for age, gender, area, daily energy intake total activity and smoking status whereas in subjects with low stress, BMI was higher in heterozygotes than major allele (Fig. 2b).

Although people with high levels of stress and MC4R minor allele had higher energy intake, it was not significantly different (Table 5). The daily intake and the percentage of fat and protein based on energy intake were also higher in people with high stress and MC4R minor allele but it was not significantly different (Table 5).

**Table 5:** Daily nutrient intake according to MC4R genotypes and stress levels

	High stress			Low stress		
	TT (*n* = 1607)	CT (*n* = 1091)	CC (*n* = 193)	TT (*n* = 3426)	CT (*n* = 2081)	CC (*n* = 347)
Energy intake (kcal)	1949 ± 599	1945 ± 647	1975 ± 647	1882 ± 661	1903 ± 684	1903 ± 970
CHO (g)	336 ± 97	337 ± 104	340 ± 109	330 ± 110	334 ± 109	327 ± 154
Protein (g)	68.2 ± 27.1	67.6 ± 30.0	69.7 ± 27.8	64.9 ± 28.5	65.7 ± 32.1	67.3 ± 37.9
Fat (g)	34.4 ± 19.8	33.8 ± 20.8	35.1 ± 20.0	31.2 ± 20.0	31.5 ± 20.5	33.9 ± 29.0
Na (mg)	3271 ± 1710	3296 ± 1750	3253 ± 1537	3111 ± 1661	3143 ± 1669	3179 ± 1951
Percentage of energy intake based on EER	91.0 ± 29.5	90.3 ± 26.7	93.1 ± 31.6	90.5 ± 33.8	91.8 ± 33.1	92.7 ± 46.4
Percentage of CHO intake based on energy intake	69.6 ± 6.6	70.0 ± 6.5	69.3 ± 6.4	70.8 ± 6.8	70.8 ± 6.5	69.8 ± 7.5
Percentage of protein intake based on energy intake	13.8 ± 2.3	13.7 ± 2.3	14.0 ± 2.2	13.6 ± 2.4	13.6 ± 2.3	13.9 ± 2.4
Percentage of fat intake based on energy intake	15.4 ± 5.2	15.1 ± 5.1	15.6 ± 5.0	14.3 ± 5.3	14.3 ± 5.0	15.1 ± 5.9

*CHO* carbohydrate, *EER* energy estimated requirement

## Discussion

Many studies have indicated that the expression of MC4R in the hypothalamus leads to excessive energy intake [15, 16], and also that it is regulated by stress through the HPA axis [22]. It has also been reported that MC4R variants are associated with the incidence of obesity [5, 10, 12]. We hypothesized that there is an interaction between MC4R rs17782313 variants and diet and lifestyle that influence the risk of obesity. There was a positive interaction between MC4R variants and mental stress levels that was significantly associated with the risk of obesity after adjusting for age, gender, residence area, daily energy intake, smoking status and physical activity. In subjects with high stress, those with MC4R minor alleles had higher BMIs after adjusting for confounders without modulating energy and nutrient intake; they also had a preference for spicy taste. Furthermore, in the group with energy intake higher than EER, subjects with MC4R minor alleles had higher BMIs than those with the major alleles but with similar energy intakes. Therefore, MC4R variants interacted with energy intake and mental stress levels to promote obesity. To the best of our knowledge, no previous studies have investigated the interactions between MC4R variants and nutrient intake and mental stress in Korean adults.

In several populations, the MAF of MC4R rs17782313 varies from 24 % to 26 %; Therefore, the MAF of MC4R rs17782313 in Koreans (25 %) was comparable to studies in other ethnicities [11, 14, 16]. Those previous studies found that the MC4R rs17782313 C allele was a risk allele for obesity. The MC4R rs17782313 CC genotype has a strong positive association with BMI which has been clearly demonstrated in European and Tatar populations in which subjects with the rs17782313 variant had higher BMIs [11, 14]. These results suggest that the MC4R minor allele is a risk factor for obesity across ethnicities. In the Korean population, people with the MC4R minor allele exhibit a small but significant increase in BMI (0.5 ± 0.04 kg/m^2^) in comparison to the MC4R major allele, but in European population the BMI difference between MC4R genotypes is 4.1 ± 9.1 kg/m^2^ [36]. The differences in BMI between MC4R genotypes were higher in the participants with high levels of stress (0.78 ± 0.05 kg/m^2^). However, it was still a much lower difference in Koreans than in Europeans [36] and Native Americans [37]. The small difference in BMI in the Korean population was associated with participants within a normal range of BMI (24.1 ± 3.1 kg/m^2^) in comparison to European population (31.8 ± 9.5 kg/m^2^). In addition, the average caloric intake of Koreans was also mostly within the EER. The higher BMI in subjects with the MC4R minor alleles was related to a small increase in daily energy intake and high fat intake in the present study. Even though daily energy intake was slightly and insignificantly higher in participants with MC4R minor alleles, it influenced the increase in BMI. In the participants with higher than EER, the participants with MC4R minor alleles had much higher BMI than those with major alleles. The present study demonstrated that MC4R rs17782313 minor allele increased BMI in participants with energy intake higher than EER. Thus, the greater increase of BMI in MC4R minor allele in European population may be involved in higher energy intake, and the less pronounced effect of the minor alleles on the BMI of Koreans may be due to overall lower BMIs and lower energy intakes in the Korean population.

Gene-environment interactions play an important role in the etiology of obesity. MC4R variants have been implicated in the modulation of nutrient intake in previous studies [21, 22]. Some studies have indicated a positive association between the MC4R rs17782313 variant and the consumption of higher-energy and fatty foods [21]. An NHS cohort study indicated a positive association between the MC4R rs17782313 variant and the consumption of higher-energy and fatty foods in the United States using FFQ [11] and multiple 1-week dietary records, and the same result was confirmed in Iran based on a 3-day food record [21]. However, Qi et al. [21] have shown that the MC4R C allele is significantly associated with high intakes of total energy (*P* = 0.028), total fat (*P* = 0.008) and protein (*P* = 0.003) after adjustment for age, BMI, and diabetes status; although the associations between MC4R rs17782313 and BMI were significant (*P* = 0.002) independent of dietary intakes. However, in the present study, the daily energy intake was not significantly different according to MC4R genotypes, but subjects with MC4R C alleles had higher intakes of processed foods than those with T alleles. This was consistent with the results that subjects with MC4R C alleles had higher fat percent based on energy intake than others. Consistent with the present study, daily energy, carbohydrates, protein and fat intakes were not significantly different among MC4R genotypes [23]. However, unlike the present study, MC4R genotype has a positive association with intake of energy, fat and protein [21]. Therefore, the association between MC4R genotypes and food intake remains unclear and further multi-center studies are needed.

No studies have reported interactions between MC4R genotypes and mental stress. We found new evidence that the MC4R rs17782313 C allele interacts with mental stress to promote obesity; but in this population, only in subjects with high stress. Some animal studies have demonstrated a relationship between MC4R gene, mental stress and food intake through the HPA axis [24–26]. MC4R facilitates the regulation of the HPA axis in response to psychological stress [26]. In MC4R knockout mice the plasma ACTH and corticosterone levels are significantly lower in response to restraint stress, suggesting that MC4R is a mediator of communication between brain and peripheral stress system to facilitate mental stress [25, 38]. Infusion of a selective MC4R agonist into the medial amygdala elicits anxiety in the elevated plus-maze test and decreases food intake [25]. In contrast, an MC4R antagonist infusion blocks restraint stress-induced anxiogenic and anorectic effects [26]. These results suggest that acute stress decreases food intake via the HPA axis. Saegusa et al. [38] reported that MC4R stimulation by stress results in decreased peripheral ghrelin concentrations, thereby suppressing food intake in animals. However, long-term stress increases serum glucocorticoid levels to induce food intake. Thus, MC4R genotypes may influence eating behaviors through stress response and may be involved in weight gain in humans.

Mental stress influences eating choices but people with mental stress have different choice of comfort food [39]. People differ in their preferences for spicy, oily, or sweet foods as comfort foods when stressed [40]. High stress situations change eating patterns and increase the consumption of highly palatable foods, which in turn augments incentive salience of highly palatable foods. Thus, the alteration of eating patterns enhances risk of weight gain and obesity [41]. Emotional eaters with stress consume more sweet foods and oily foods than unstressed and non-emotional eaters [40]. Participants with a high level of work stress consumed more saturated fat and high-energy foods [42, 43]. Korean high school students consumed more sweet foods with mental stress [43]. Thus, the choice of comfort foods is somewhat different according to age, gender, and personality, but may also be related to differences in genetic backgrounds. In the present study, subjects with MC4R minor alleles consumed a higher percentage of processed foods, which might be related to a busy lifestyle but not the preference of taste.

There were several limitations to our analysis. First, cause-and-effect cannot be established because this was a cross-sectional cohort study. Second, obese participants with the rs17782313 CC genotype were underrepresented (*n* = 544). Third, preferences for particular foods were not strongly correlated with food intake as measured by the FFQ. There are 103 kinds of foods in the FFQ, but only 5 spicy foods (cabbage kimchi, radish kimchi, nabak kimchi, green pepper, and onion), and spiciness is not dependent on the kind of food but rather on the amount of red pepper used. Thus, the preference for spicy foods cannot be matched with the results of the FFQs. Only green peppers can be said to partly reflect the preference of spiciness but this pepper is not frequently consumed. However, the preference of sour taste and fruit intake was somewhat consistent: subjects with the MC4R minor allele consumed less fruits and subjects with MC4R minor allele did not prefer sour taste in low stress state. Nonetheless, the consumption of green peppers tended to increase with the MC4R C allele but the association was not significant. Thus, MC4R variants were positively associated with a preference for spicy foods. The FFQ included seven processed foods: cheese, ramen, frozen dumplings, sausage, chips, canned tuna, and ham. The preference for processed foods may be well reflected by the FFQ. However, Brazilian subjects who consumed more processed foods did not consider processed foods to be a preference, but they preferred to consume oily foods [44].

## Conclusions

We confirmed that there was a positive interaction between MC4R variants and energy intake which was associated with increased risk of obesity after adjusting for confounders. The interaction of mental stress with MC4R significantly increased the risk of obesity: Korean adults with MC4R minor alleles had a higher risk of obesity in high stress states independent of other obesity related factors. Furthermore, Korean adults with C alleles had increased risk of obesity only with energy intakes in excess of the EER. Therefore, this research might identify subjects with specific MC4R minor alleles as a human subset of people with a low metabolic tolerance for excessive energy intake, especially when under stress.

## Abbreviations

GWAS: genome-wide association studies
FTO: fat mass and obesity-associated protein
MC4R: melanocortin-4 receptor
SNPs: single-nucleotide polymorphisms
POMC: pro-opiomelanocortin
α-MSH: α–melanocyte-stimulating hormone
AGRP: agouti-related peptide
HPA: hypothalamic-pituitary-adrenal
ACTH: adrenocorticotropic hormone
KoGES: Korean Genome Epidemiology Study
BMI: body mass index
FFQ: semi-quantitative food frequency questionnaire
EER: estimated energy requirement
DRI: dietary reference intake
SDs: standard deviations
CI: confidence intervals
